# Assessment of the direct effects of DDAH I on tumour angiogenesis in vivo

**DOI:** 10.1007/s10456-018-9617-6

**Published:** 2018-05-02

**Authors:** Efthymia Papaevangelou, Jessica K. R. Boult, Guy S. Whitley, Simon P. Robinson, Franklyn A. Howe

**Affiliations:** 1 Molecular and Clinical Sciences Research Institute, St. George’s, University of London, Cranmer Terrace, London, SW17 0RE UK; 2 Division of Radiotherapy and Imaging, The Institute of Cancer Research, 15 Cotswold Road, Sutton, Surrey SM2 5NG UK; 3 School of Immunology and Microbial Sciences, Guy’s Hospital, King’s College London, London, UK

**Keywords:** DDAH, Nitric oxide, Inducible expression, MRI, Tumour angiogenesis

## Abstract

Nitric oxide (NO) has been strongly implicated in glioma progression and angiogenesis. The endogenous inhibitors of NO synthesis, asymmetric dimethylarginine (ADMA) and *N*-monomethyl-l-arginine (l-NMMA), are metabolized by dimethylarginine dimethylaminohydrolase (DDAH), and hence, DDAH is an intracellular factor that regulates NO. However, DDAH may also have an NO-independent action. We aimed to investigate whether DDAH I has any direct role in tumour vascular development and growth independent of its NO-mediated effects, in order to establish the future potential of DDAH inhibition as an anti-angiogenic treatment strategy. A clone of rat C6 glioma cells deficient in NO production expressing a pTet Off regulatable element was identified and engineered to overexpress DDAH I in the absence of doxycycline. Xenografts derived from these cells were propagated in the presence or absence of doxycycline and susceptibility magnetic resonance imaging used to assess functional vasculature in vivo. Pathological correlates of tumour vascular density, maturation and function were also sought. In the absence of doxycycline, tumours exhibited high DDAH I expression and activity, which was suppressed in its presence. However, overexpression of DDAH I had no measurable effect on tumour growth, vessel density, function or maturation. These data suggest that in C6 gliomas DDAH has no NO-independent effects on tumour growth and angiogenesis, and that the therapeutic potential of targeting DDAH in gliomas should only be considered in the context of NO regulation.

**Electronic supplementary material:**

The online version of this article (10.1007/s10456-018-9617-6) contains supplementary material, which is available to authorized users.

## Introduction

Angiogenesis is an important driver of the growth and development of solid tumours. Several angiogenic stimuli also induce the production of nitric oxide (NO). Strong positive correlations between the expression of nitric oxide synthases (NOS) and tumour progression have been identified in a number of human cancers [1]. Intracellular factors which regulate NO synthesis may therefore represent important targets in the control of tumour growth and vascularization.

The synthesis of NO can be modulated by the two dimethylarginine dimethylaminohydrolase (DDAH) isoforms via the asymmetric dimethylarginine (ADMA) pathway [2]. DDAH enzymes catalyse the hydrolysis of methylarginines, ADMA and *N*-monomethyl-l-arginine (l-NMMA), to citrulline and either dimethylamine or methylamine, respectively [3]. ADMA and l-NMMA are endogenous, competitive inhibitors of all three NOS isoforms, and NO production is physiologically regulated by methylarginines [4].

DDAH activity has been detected in various human tumours including those arising in the brain; astrocytomas in particular exhibited high levels of DDAH activity [5]. High expression of DDAH I has also been observed in aggressive triple-negative breast cancer cell lines, where DDAH I was a key regulator of cell migration and vasculogenic mimicry [6]. Overexpression of DDAH I has been shown to increase NO production and enhance tumour growth and angiogenesis in C6 gliomas in vivo [7, 8]. Pharmacological manipulation of DDAH may be an attractive therapeutic target for regulating NO in cancer. DDAH inhibitors can help overcome one of the major considerations for NO-focused therapies, which is targeting pathological NO production while sparing beneficial NO produced constitutively. Direct non-selective NOS inhibitors can lead to inherent adverse effects by limiting the NO antimicrobial properties or by inhibiting the constitutive NOS isoforms [9].

Previous studies have looked into DDAH-mediated NO-dependent effects on tumour vascularization. However, other studies have also suggested that DDAH and/or ADMA can exert direct, NO-independent effects on angiogenesis. For example, DDAH II overexpression led to increased vascular endothelial growth factor (VEGF) expression in endothelial cells by binding to protein kinase A (PKA), increasing phosphorylation and activation of the transcription factor Sp1 [10, 11]. These studies imply that the DDAH/ADMA pathway may directly regulate tumour angiogenesis. We can hypothesize that if DDAH has NO-independent effects, then it might be a better anti-cancer target than NO synthases, as NO operates in a bimodal fashion in cancer and can be both pro- and anti-tumorigenic, depending on its concentration and the tumour microenvironment [12, 13].

To investigate whether there are any NO-independent effects of tumour-derived DDAH I on tumour angiogenesis, we first identified a C6-derived glioma cell line deficient in NO production, and genetically modified it to overexpress DDAH I under doxycycline (DOX) regulation. These cells were then characterized and compared against parental and wild-type cells in vitro. Subsequently, the vascular phenotype of tumours derived from the regulatable cells was compared in the presence or absence of DOX and with tumours derived from constitutive DDAH I overexpressing NO-producing C6 cells. The vascular functionality of these tumours was interrogated in vivo using quantitative non-invasive MRI, and ex vivo histopathological analysis.

## Materials and methods

### Cell culture

Both wild-type and transfected C6 rat glioma cells (European Collection of Cell Cultures, Salisbury, UK) were maintained in Nutrient Ham’s F-10 (Sigma-Aldrich, Dorset, UK) medium containing 2 mM l-glutamine, 100 U/ml penicillin, 0.2 mg/ml streptomycin and 10% (v/v) tetracycline-free fetal bovine serum (Clontech, Saint-Germain-en-Laye, France).

To achieve inducible overexpression of DDAH I, C6 wild-type (wt) cells were engineered to include a DDAH I cDNA sequence combined with a tetracycline-inducible system (pTet Off), which allowed gene expression to be controlled by tetracycline, or as in our study, by its water soluble derivative, DOX. Briefly, C6 cells transfected with the pRevTet-Off-IN vector (Clontech), referred to as C6 parental (par) cells, were kindly provided by Prof. M. Neeman (Weizmann Institute of Science, Rehovot, Israel). These cells were selected for this study as they did not express iNOS and produced limited NO. Full length rat DDAH I cDNA (previously generated by Kostourou et al. [5]) was cloned into the BamHI site of the pTRE2hyg plasmid (Clontech) to generate pTRE2hyg.DDAHI. C6 par cells were transfected with the pTRE2hyg.DDAHI vector using Fugene 6 and selection was performed with 500 µg/ml hygromycin B in the culture medium in the presence of 1 µg/ml DOX. The stably transfected clone (designated C6 DDAH) with the highest DDAH I overexpression in the absence of DOX was chosen for further experimentation, and grown in the presence or absence of 2 µg/ml DOX. Table 1 lists all the cell lines and tumour types used in the present study and their main characteristics.

**Table 1:** List of cell lines and tumours used in the present study and their main characteristics

In vitro experiments				
Cell line name	C6 wt	C6 parental	C6 DDAH	
**Description**	Wild-type C6 cells	C6 cells with pTet-Off element (parental to C6 DDAH cells)	DOX-inducible DDAH I overexpressing C6 cells	
**DOX**	Independent	Independent	Absence (− DOX)	Presence (+ DOX)
**DDAH I expression**	Low endogenous expression	Low endogenous expression	Overexpression	Low endogenous expression
**NO production**	Increased by cytokines	Negligible (cytokine independent)	Negligible (cytokine independent)	Negligible (cytokine independent)
**VEGF expression**	Endogenous	Endogenous	Low	Low

In vivo experiments					
Tumour type	C6 DDAH group A (from cells grown in normal medium)		C6 DDAH group B (from cells grown in medium with DOX)		D27
**Description**	DOX-inducible DDAH I overexpressing C6 cells		DOX-inducible DDAH I overexpressing C6 cells		Constitutively DDAH I overexpressing C6 cells
**DOX**	Absence (− DOX)	Presence (+ DOX)	Absence (− DOX)	Presence (+ DOX)	Independent
**DDAH I expression**	Overexpression	Low endogenous expression	Overexpression	Low endogenous expression	Overexpression
**NO production**	Negligible	Negligible	Negligible	Negligible	High
**VEGF expression**	Low	Low	Low	Low	Very high

### Nitric oxide production

Cells were stimulated with cytokines (10 ng/ml TNF-α, 1000 U/ml IFN-γ) and 5 µg/ml LPS. Nitrite and nitrate production provided a proxy measure of NO synthesis. Nitrate was reduced to nitrite using vanadium chloride, and the total nitrate plus nitrite (NOx) determined using the Griess reaction [14, 15]. All experiments were performed in triplicate.

### Western blot analysis

The protein concentration of cell lysates and tumour homogenates was determined using the Bradford assay [16] and equal amounts of protein were analysed by SDS-PAGE. DDAH I and iNOS expression were detected using goat polyclonal anti-mouse/rat DDAH I antibodies and rabbit polyclonal anti-mouse/rat iNOS antibodies (M-19; sc-650, Santa Cruz, Heidelberg, Germany) respectively. Polyclonal anti-α-actin antibodies (Sigma-Aldrich) or polyclonal anti-α-tubulin antibodies (Sigma-Aldrich) were used to verify equal protein loading. The integrated density of the individual bands for iNOS and DDAH I was measured and corrected using the integrated density of the individual bands for either α-actin or α-tubulin using ImageJ software [17].

### DDAH activity assay

DDAH I activity was determined by measuring the conversion of ADMA to l-citrulline [18]. Lyophilized cells (200 µg protein) were mixed with 20 µl 4 mM ADMA in phosphate-buffered saline (PBS, pH 6.5) and incubated for 4 h at 37 °C. The enzymatic reaction was stopped using an equal volume of 10% (w/v) trichloroacetic acid. Samples were centrifuged and the supernatants were mixed with a 2:1 ratio mixture of antipyrine (0.5% (w/v) in 50% sulphuric acid) and diacetyl monoxime (0.8% (w/v) in 5% acetic acid). Subsequently, samples were heated in a boiling water bath for 30 min and the amount of l-citrulline formed was determined by measuring the absorbance at 466 nm. To detect DDAH I activity in tumours, tumour homogenates (400 µg of protein) were initially incubated with 4 µl of 2500 U/ml Urease from *Canavalia ensiformis* for 20 min at 37 °C in order to eliminate excess urea [19]. Subsequently, samples were mixed with 25 µl 4 mM ADMA substrate in PBS and incubated for 20 h at 37 °C. The rest of the assay was performed in a similar manner as for the lysed cells. The enzymatic activity was corrected for protein concentration.

### VEGF ELISA

VEGF concentration was measured in cell culture medium, collected from confluent 3 cm dishes 72 h after seeding, and in tumour homogenates using a rat VEGF ELISA kit (PeproTech, London, UK) according to manufacturer’s instructions. Values were normalized to protein concentration.

### Animals and tumours

Experiments were performed in accordance with the local ethical review panel, the UK Home Office Scientific Procedures Act 1986 and the UK National Cancer Research Institute Guidelines for the Welfare and Use of Animals in Cancer Research [20]. Female (7–8 weeks old) NCr nude mice were injected subcutaneously in the flanks with 2 × 10^6^ cells in 0.1 ml PBS. Tumour volume was calculated using the ellipsoid shape formula: (π/6) × *Length* × *Width* × *Depth*. Tumour doubling times (TDT) were calculated based on the individual tumour growth curves on a logarithmic plot using the formula: TDT = ln(2)/[slope of growth curve]. Prior to implantation, C6 DDAH cells were pre-treated for 5 days with DOX (C6 DDAH group A) or were grown in normal medium without DOX (C6 DDAH group B). Animals injected with C6 DDAH cells (groups A and B) were given drinking water containing 5% (w/v) sucrose with or without 0.2 mg/ml DOX (*n* = 6 per group) (C6 DDAH ± DOX group A and C6 DDAH ± DOX group B). Additional animals (*n* = 4) were injected with constitutively DDAH I overexpressing cells (clone D27), previously engineered and characterized by Kostourou et al [5].

### Magnetic resonance imaging

Mice bearing size-matched (~ 500 mm^3^) tumours were anaesthetised with a 10 ml/kg intraperitoneal injection of Hypnorm (0.315 mg/ml fentanyl citrate plus 10 mg/ml fluanisone; Janssen Pharmaceutical, Wantage, UK), Hypnovel (5 mg/ml midazolam; Roche, West Sussex, UK) and water (1:1:2), and positioned so the tumour hung within a three-turn 25-mm-diameter surface coil for MRI using a 4.7 T Varian Unity INOVA horizontal small-bore imaging system. The mouse core temperature was maintained at 37 °C using heated air blown through the magnet bore. Blood oxygen saturation was monitored using a MouseOx Pulse Oximeter (Braintree Scientific, MA, US).

T_2_-weighted spin echo images were acquired from seven axial 1-mm-thick slices positioned across the whole tumour, using a repetition time (TR) of 1500 ms, an echo time (TE) of 30 ms, and a 128 × 128 matrix over a 2.56-cm field of view. Intrinsic susceptibility MRI was performed to assess vessel function and maturation, utilizing carbogen (95% O_2_/5% CO_2_) breathing to increase blood oxygenation and localised vascular smooth muscle dilation. The changes in the tumour transverse relaxation rate *R*_2_* (s^−1^) caused by perturbations in the paramagnetic deoxyhaemoglobin in the blood vessels were measured using a multi-gradient echo (MGRE) sequence. MGRE images were acquired from seven slices with TR of 450 ms, TE of 7–56 ms, an echo spacing of 7 ms and flip angle (α) of 45° during air and following a 5-min transition period during carbogen (95% O_2_/5% CO_2_) breathing [21–23].

Susceptibility contrast MRI was then performed to quantify the tumour fractional blood volume (fBV, %). MGRE images were acquired, 5 min after air breathing was resumed, prior to and 5 min after intravenous injection of 5.2 mgFe/kg of the ultrasmall superparamagnetic iron oxide (USPIO) contrast agent ferumoxtran (Guerbet S.A., Villepinte, France). USPIO particles were used as a blood pool contrast agent that creates magnetic susceptibility variations close to blood vessels leading to an increase in water *R*_2_* in the surrounding tissue [24].

### MRI data analysis

*R*_2_* maps were calculated on a voxel-by-voxel basis from MGRE image data using ImageJ and Matlab. Average apparent *R*_2_* relaxation rates were calculated for each slice for a region of interest (ROI), defined from the associated T_2_-weighted image, encompassing the whole tumour but excluding the surrounding skin and muscle. Carbogen-induced changes in R_2_* (Δ*R*_2_*_CB_ = *R*_2_*_carbogen_ − *R*_2_*_air_) were determined over the whole tumour. Tumour fBV was determined over the same ROI from the increase in R_2_* (Δ*R*_2_*_USPIO_ = *R*_2_*_post−USPIO_ − *R*_2_*_pre−USPIO_) caused by the USPIO particles as previously described [24, 25].

### Histological analysis and microscopy

Following the MRI, mice were administered intraperitoneally with 60 mg/kg of the hypoxia marker pimonidazole hydrochloride (Hypoxyprobe, Burlington, MA, USA) in PBS. After 45 min, mice were also injected intravenously with 15 mg/kg of the perfusion marker Hoechst 33342 (Sigma-Aldrich, Dorset, UK) in PBS. Tumours were excised after 1 min and snap-frozen. For each tumour, three acetone-fixed cryosections (10 µm) were visualized for uptake of Hoechst 33342 by fluorescence microscopy using a motorized scanning stage (Prior Scientific Instruments, Cambridge, UK) attached to a BX51 microscope (Olympus Optical, London, UK) driven by CellP (Soft Imaging System, Munster, Germany) to record composite digital images of whole tumour sections. The same sections were then processed for pimonidazole adduct formation using Hypoxyprobe-1 plus FITC-conjugated mouse monoclonal antibodies and imaged using the same stage coordinates. To assess endothelial and perivascular cell content, additional sections were stained with rat monoclonal anti-mouse CD31 antibodies [MEC 7.46] (ab7388, Abcam, Cambridge, UK), biotinylated goat anti-rat immunoglobulins (IgG) (Vector Laboratories, Peterborough, UK) and Fluorescein Avidin D (Vector Laboratories), and with rabbit polyclonal antibodies against smooth muscle actin (α-SMA, ab5694, Abcam) and Alexa-Fluor 546 goat anti-rabbit secondary antibodies. The cell nuclei were counterstained with DAPI (4′,6-diamidino-2-phenylindole). Non-immune mouse and rabbit IgG antibodies were used in the same concentration with CD31 and α-SMA antibodies respectively as negative isotype controls. Composite images were acquired using fluorescence microscopy as previously described [26]. Tumour sections were also stained with haematoxylin and eosin (H&E), and composite images were acquired under light microscopy.

Post-processing was performed on composite digital images using ImageJ software [17]. ROIs of whole tumour sections were defined and fluorescent particles detected above a constant colour threshold across all sections, which was higher than background fluorescence defined from isotype control stained tumour sections. The area of the tumour section with fluorescence was determined and expressed as a percentage of the whole tumour section, and for each tumour the average across three slices was determined. ROIs were also drawn around all necrotic foci on H&E-stained sections and necrosis was expressed as a percentage averaged across three tumour slices for each tumour.

### Statistical analysis

Data were analysed using GraphPad Prism (GraphPad Software Inc., La Jolla, CA, USA). Results are presented as mean ± 1 standard error of the mean (SEM) with significance testing at a 5% confidence level. Student’s unpaired *t-*test was used to compare two groups and repeated measures ANOVA with Bonferroni’s Multiple Comparison post-test was used to compare multiple groups.

## Results

### NO production is limited in C6 DDAH cells

**Fig. 1:**
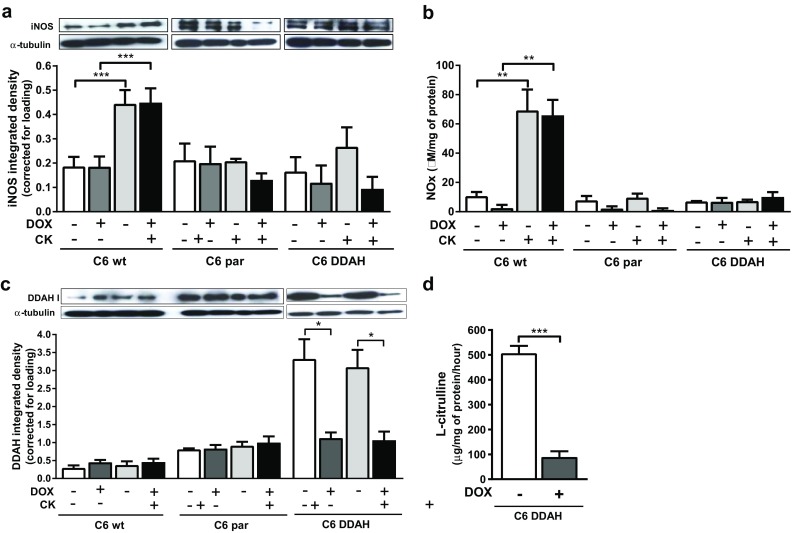
In vitro characterization of C6 DDAH cells in comparison with parental cells. Cells were treated for 4 days with 2 µg/ml DOX. For the last 48 h, cells were also stimulated with cytokines (10 ng/ml TNF-α, 1000 U/ml IFN-γ) and 5 µg/ml LPS. **a** iNOS (131 kDa) and **c** DDAH I (34 kDa) expression determined by western blot and integrated density of individual bands (corrected for loading). **b** NOx production. **d** l-citrulline production evaluated after 4-h incubation with ADMA substrate. Results are mean + 1 SEM of three separate experiments (*P < 0.05, **P < 0.01, ***P < 0.001)

Initial experiments confirmed that the basal production of NO by C6 parental cells was negligible and that they did not express iNOS in response to combined stimulation with cytokines and LPS (cytokine independent). As anticipated, iNOS expression was also unaffected by cytokine and LPS stimulus and production of NOx was low in C6 DDAH cells, which were derived from the selected C6 parental cells. In contrast, cytokine stimulation of C6 wild-type cells led to increased iNOS protein expression (2.3-fold) and NOx production (Fig. 1a, b; Table 1). The presence of DOX did not affect iNOS expression or NO synthesis in the C6 wt, C6 par or C6 DDAH cell lines.

### DDAH I overexpression occurs in the absence of DOX in C6 DDAH cells

Expression of DDAH I protein was low (endogenous expression) in C6 wild-type and C6 parental cells and expression remained unaffected by the presence of DOX (Fig. 1c; Table 1). C6 DDAH cells exhibited a tight in vitro regulation of DDAH I expression with DOX. In the absence of DOX, the cells expressed high levels of active DDAH I enzyme from the pTRE2hyg.DDAHI vector, which in turn led to high l-citrulline production. In the presence of DOX, only endogenous DDAH I was expressed, whereas removal of DOX led the overexpression of DDAH I, resulting in a fivefold increase in activity (Fig. 1d; Table 1). Overexpression of DDAH I did not affect the growth properties of cells in vitro; cell proliferation and death over 5 days were the same in the presence or absence of DOX (Supplementary Fig. 1a, b). Cytokines and LPS had no effect on the expression of DDAH I by all three cell lines (Fig. 1c).

### DDAH I up-regulation in tumours does not affect growth rate when NO is absent

**Fig. 2:**
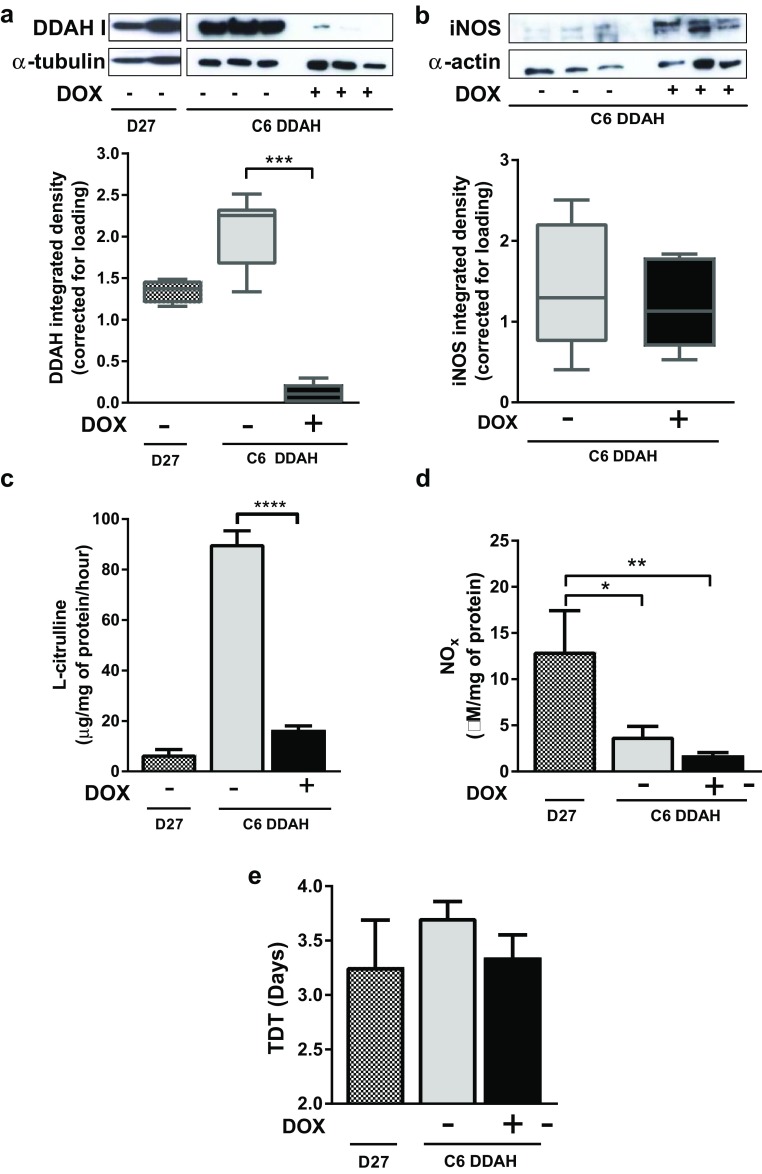
In vivo characterization of C6 DDAH tumour xenografts compared with D27 tumour xenografts. C6 DDAH cells were pre-treated with 2 µg/ml DOX in the medium for 5 days (group A). Mice with C6 DDAH tumours were given 0.2 mg/ml DOX in 5% (w/v) sucrose or 5% (w/v) sucrose alone (*n* = 6 per group) in the drinking water ad libitum. Mice with D27 (C6 constitutively DDAH overexpressing) tumours were given drinking water alone (*n* = 4). **a, b** Example western blots of tumour homogenates and mean box plot distributions of the integrated densities of the individual bands for DDAH I (**a**) and iNOS (**b**) corrected using α-tubulin and α-actin respectively. **c** l-citrulline production and (**d**) NOx production by tumours, determined from homogenates. **e** TDTs in days. Results are the mean + 1 SEM of measurements made from all tumours in each group (*P < 0.05, **P < 0.01, ***P < 0.001, ****P < 0.0001, One-Way ANOVA with Bonferroni’s multiple comparison post-test)

To investigate whether overexpression of DDAH I in tumours affects growth rate in the absence of NO, tumours derived from C6 DDAH cells were grown subcutaneously in mice and compared with constitutive DDAH I overexpressing tumours (D27), in which the DDAH/ADMA/NO pathway is fully functional. Results from mice with C6 DDAH tumours derived from cells pre-treated for 5 days with DOX (group A) are presented in Fig. 2 together with results from mice with D27 tumours. Results from mice with C6 DDAH tumours derived from cells grown in normal medium without DOX (group B) were very similar to results from group A and therefore are presented in Supplementary Fig. 2. DDAH I protein expression in C6 DDAH (groups A and B) tumours in mice not given DOX in the drinking water was comparable with expression in D27 tumours. However, addition of DOX in the drinking water was sufficient to “switch off” DDAH I overexpression in C6 DDAH tumours (both groups) returning to the low endogenous DDAH I expression levels (Fig. 2a, Supplementary Fig. 2a, Table 1). Production of l-citrulline, and hence DDAH enzymatic activity, was approximately five times higher in C6 DDAH tumours grown without DOX compared with those grown with DOX in both groups A and B (Fig. 2c, Supplementary Fig. 2b). Unexpectedly, D27 tumours had significantly lower levels of DDAH activity (~ 18-fold) than C6 DDAH − DOX tumours (group A) (Fig. 2c). Pre-treatment of the C6 DDAH cells with DOX prior to implantation (group A) had no effect on DDAH I activity. No significant differences were observed in the expression of iNOS or the production of NOx between C6 DDAH ± DOX tumours (group A) (Fig. 2b, d; Table 1). In contrast, NOx production was significantly higher (threefold) in D27 tumours compared with the C6 DDAH ± DOX tumours (group A). DDAH I overexpression in the absence of NO had no effect on the growth rate of C6 DDAH tumours (group A) (Fig. 2e, Supplementary Fig. 1c). Pre-treating the cells with or without DOX also had no effect on the growth rate of C6 DDAH tumours (groups A and B) (Fig. 2e, Supplementary Fig. 2c). All tumours grew with similar growth rates, with TDTs between 3 and 4 days.

### DDAH I overexpression does not alter VEGF production when NO is limited

**Fig. 3:**
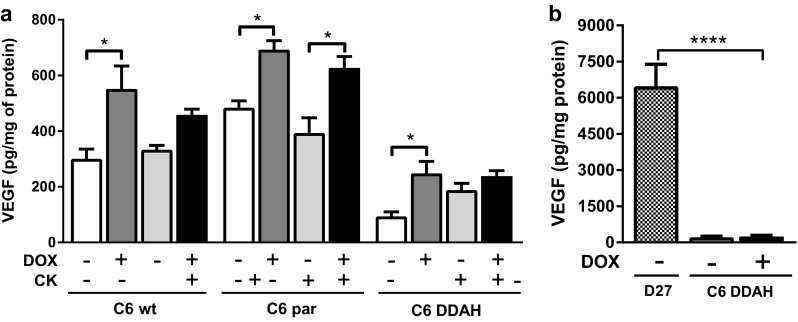
Investigation of the effect of DDAH I on VEGF expression in vitro and in vivo. **a** VEGF levels in culture medium from cells treated with 2 µg/ml DOX for 6 days. For the last day, cells were also stimulated with cytokines (10 ng/ml TNF-α, 1000 U/ml IFN-γ) and 5 µg/ml LPS. Culture medium was collected from the last 24 h of treatment. **b** VEGF expression in tumour homogenates. C6 DDAH tumours (group A) derived from animals provided with drinking water containing 0.2 mg/ml DOX in 5% (w/v) sucrose or 5% (w/v) sucrose alone (*n* = 6 per group). Mice with D27 tumours were given drinking water alone (*n* = 4). Results are mean + 1 SEM of duplicate measurements from three separate in vitro experiments or from all tumours in each group (*P < 0.05, ****P < 0.0001, One-Way ANOVA with Bonferroni’s multiple comparison post-test)

Having characterised the DDAH/ADMA/NO axis in the C6 DDAH cells and tumours, the effects of DDAH I overexpression on VEGF were investigated. C6 DDAH ± DOX cells produced less VEGF than C6 wt and C6 par cells, and DDAH I overexpression in the C6 DDAH − DOX cells did not increase VEGF production (Fig. 3a; Table 1). Moreover, cytokine stimulation did not have an effect on VEGF production in C6 wt, C6 par or C6 DDAH cells in the absence or presence of DOX. Interestingly, the presence of DOX increased VEGF production by 1.8-fold in C6 wt cells, 1.5-fold in C6 par and 2.5-fold in C6 DDAH cells (Fig. 3a). In tumour homogenates, VEGF production was low in all C6 DDAH tumours (groups A and B) and no significant differences were found between tumours grown in the presence or absence of DOX (Fig. 3b, Supplementary Fig. 2d, Table 1). VEGF production was markedly higher (~ 26-fold) in D27 tumours compared with C6 DDAH ± DOX tumours (group A). Results for DDAH I expression, NO production and VEGF expression from all cell lines and tumours are briefly summarized in Table 1.

### In the absence of NO tumour vascular development is unaffected by DDAH I up-regulation

**Fig. 4:**
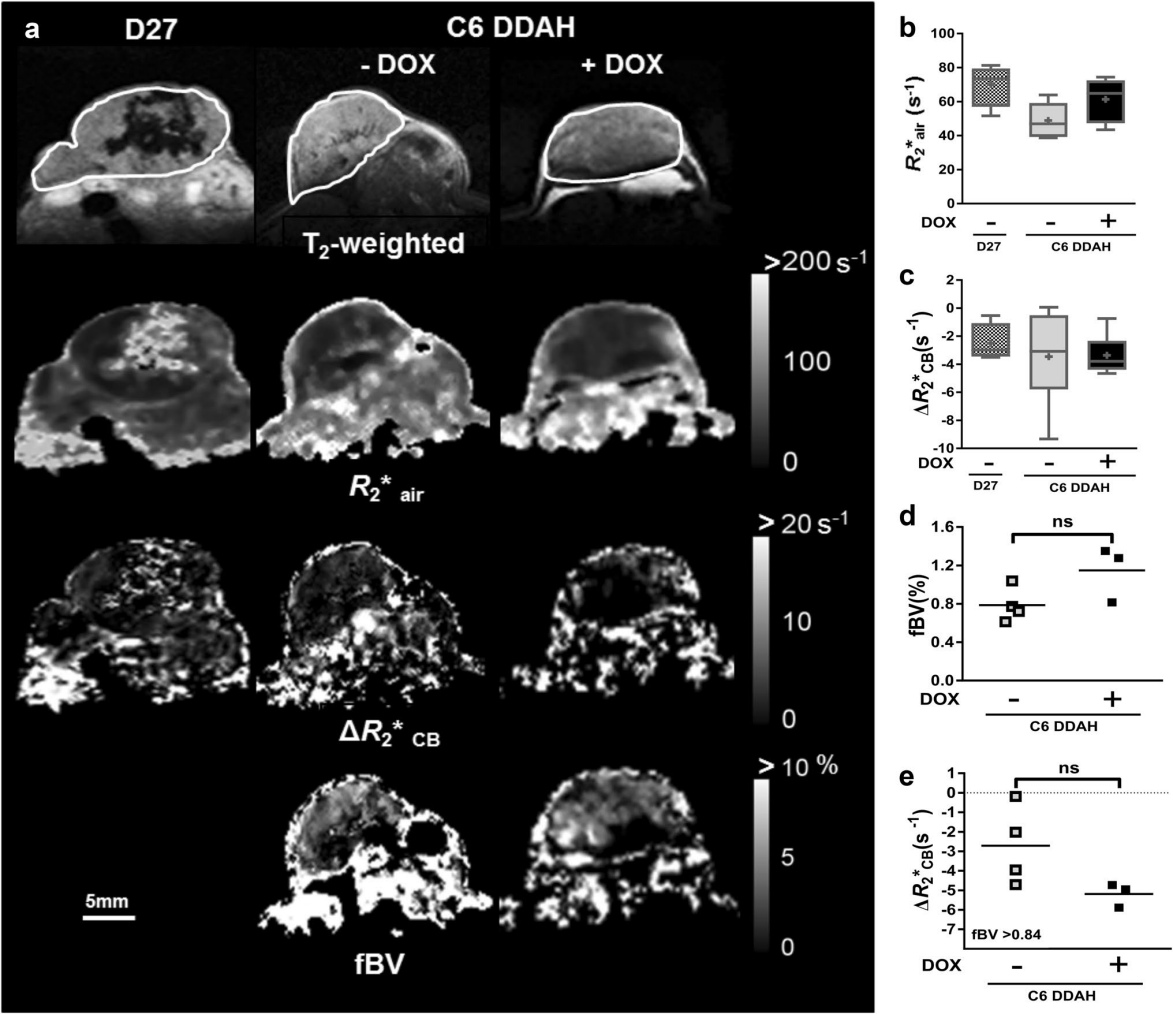
The effects of DDAH I up-regulation on tumour vascular development as determined by MRI of C6 DDAH xenografts (group A). **a** Representative T_2_-weighted images, parametric maps of baseline *R*_2_*_air_, *ΔR*_2_*_CB_ in response to carbogen and fractional blood volume (fBV) acquired from the central tumour slice. ROIs encompassing the whole tumour volume are demarcated on the T_2_-weighted images. Box plot distributions of the median *R*_2_*_air_ (**b**) and *ΔR*_2_*_CB_ (**c**) values measured over the whole tumour volume of D27 (*n* = 4) and C6 DDAH ± DOX (*n* = 6) xenografts. Median fBV values (**d**) and median *ΔR*_2_*_CB_ values (**e**) in regions where fBV is higher than the median fBV value of all the data of C6 DDAH − DOX (*n* = 4) and C6 DDAH + DOX (*n* = 3) tumours

Non-invasive MRI was used to explore whether DDAH I overexpression in C6 DDAH tumours (groups A and B) caused any NO-independent effects on functional tumour vasculature in vivo. Representative T_2_-weighted images and parametric maps of baseline R_2_* and carbogen-induced Δ*R*_2_* (Δ*R*_2_*_CB_) from D27 and C6 DDAH ± DOX tumours (group A) are shown in Fig. 4a. Parametric maps of fractional blood volume (fBV) are also shown for the C6 DDAH ± DOX tumours (group A). No differences in baseline *R*_2_* were found across the tumour groups (Fig. 4b, Supplementary Fig. 2e). All mice physiologically responded to carbogen breathing with an increase in blood oxygen saturation of 4 ± 1% across all groups. However, carbogen inhalation induced a negligible change, approximately  −3.2 s^−1^, in mean tumour *R*_2_* in all cohorts (Fig. 4c, Supplementary Fig. 2f). C6 DDAH tumours typically exhibited a low fBV (less than 1.5%), and despite a trend towards higher fBV in the C6 DDAH + DOX tumours (group A), the difference was not significant (Fig. 4d). Application of a retrospective analysis, previously described in Papaevangelou et al. [22], to the Δ*R*_2_*_CB_ maps, in which regions with vascular volume greater than the overall median of all the C6 DDAH tumours (fBV > 0.84%) were selected, the Δ*R*_2_*_CB_ response was smaller in C6 DDAH tumours (group A) grown without DOX compared to those grown with DOX, but this difference was not significant (Fig. 4e). No differences were observed between the baseline *R*_2_* measured during air breathing prior to carbogen challenge (*R*_2_*_air_) and that measured following resumption of air breathing prior to administration of USPIO particles (*R*_2_*_pre−USPIO_).

**Fig. 5:**
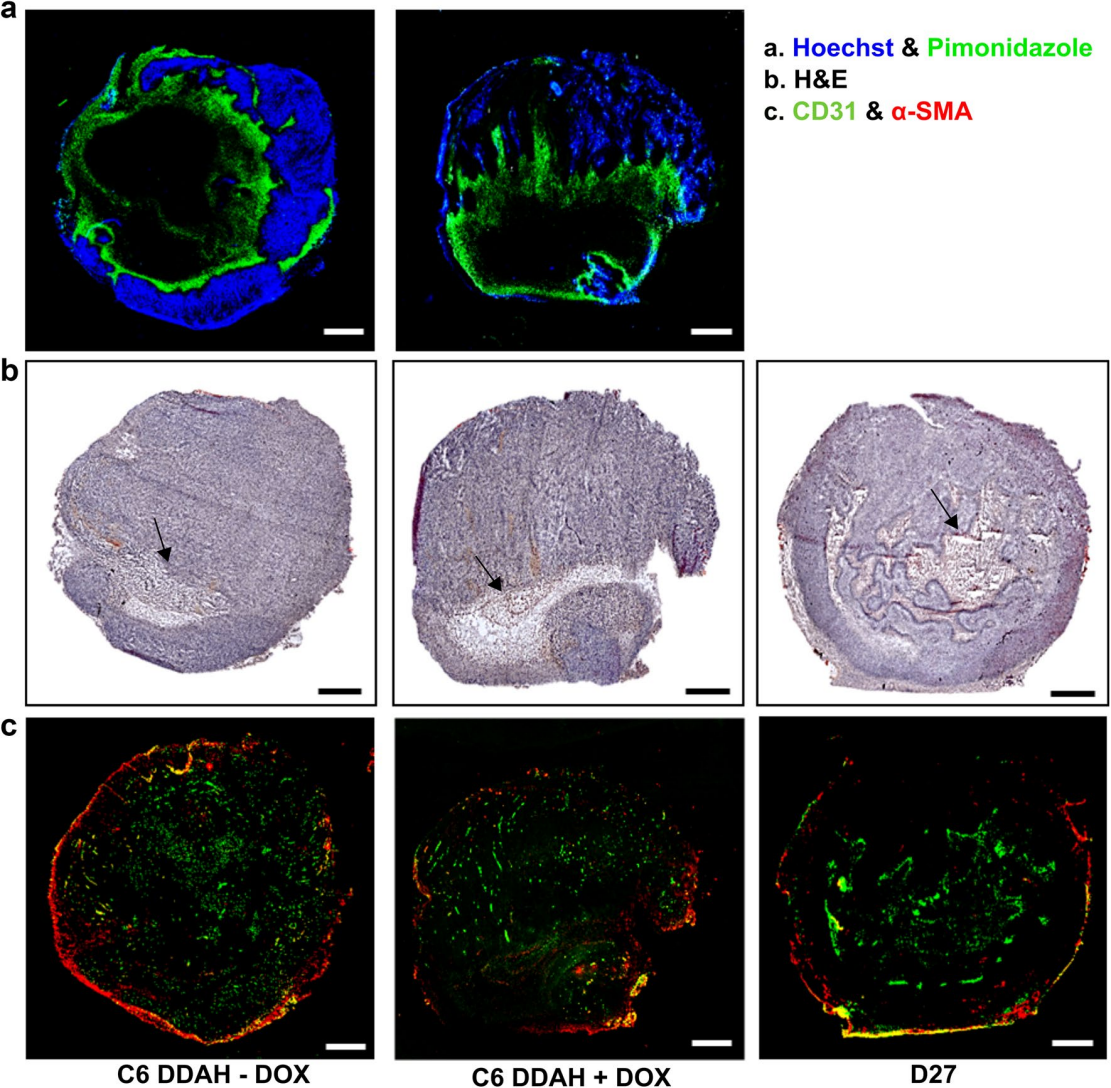
Histological assessment of the vasculature of C6 DDAH ± DOX (group A) and D27 xenografts. **a** Representative composite images from frozen whole C6 DDAH ± DOX tumour sections stained with the perfusion marker Hoechst 33342 (blue fluorescence) and the hypoxia marker pimonidazole (green fluorescence). **b** H&E-stained frozen sections from C6 DDAH ± DOX and D27 tumours indicating necrotic regions (arrows). **c** Composite images from frozen C6 DDAH ± DOX and D27 tumour sections stained with the endothelial cell marker CD31 (green fluorescence) and the perivascular cell marker α-SMA (red fluorescence). Scale bar is 1 mm

**Fig. 6:**
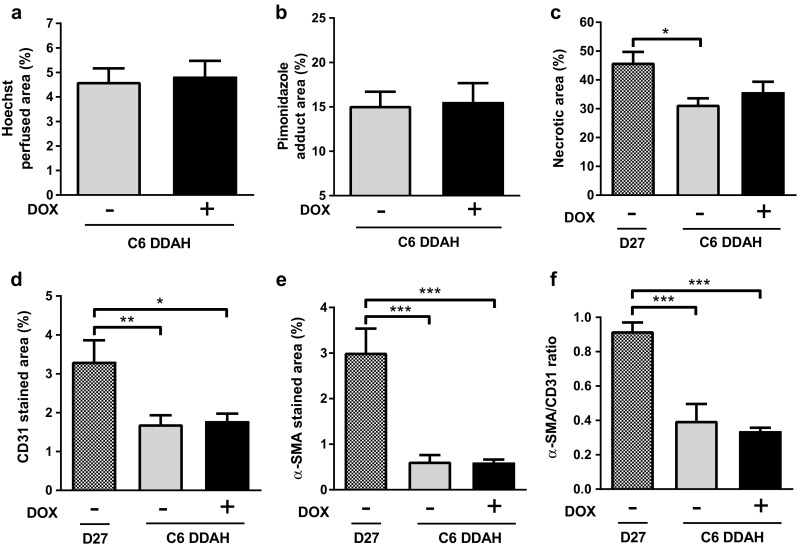
Quantification of histological markers of tumour vasculature for C6 DDAH ± DOX (group A) and D27 xenografts. **a** Hoechst 33342 perfused area, **b** pimonidazole adduct area, **c** necrotic area, **d** endothelial cell content (CD31 stained area), **e** perivascular cell coverage (α-SMA stained area), and **f** fraction of mature vessels (α-SMA/CD31 ratio) of tumour xenografts. Results are means + 1 SEM of three sections per tumour for *n* = 4 in the D27 group and *n* = 6 in the C6 DDAH ± DOX groups (*P < 0.05, **P < 0.01, ***P < 0.001)

Ex vivo histological analysis was also used to assess the tumour vasculature. Representative histological images from D27 and C6 DDAH ± DOX (group A) tumours are shown in Fig. 5, and some higher magnification images are shown in Supplementary Fig. 3. Hoechst was found closer to the periphery of the tumour where vessels were better perfused, whereas pimonidazole adducts were located closer to the tumour centre nearer to necrotic areas (Fig. 5a). More necrotic regions (indicated with black arrows) were observed in D27 tumours compared with C6 DDAH ± DOX (group A) tumours (Fig. 5b). Mature vessel smooth muscle cells and pericytes surrounded the endothelial cell layer (Fig. 5c). White arrows in the composite images indicate large vessels where the localization of CD31 and α-SMA in the vessel wall is more profound (Supplementary Fig. 3). DDAH I overexpression did not result in any differences in the degree of tumour perfusion (Hoechst perfused area) and hypoxia (pimonidazole adduct area) in the C6 DDAH ± DOX tumours (group A) (Fig. 6a, b). The extent of necrosis was significantly higher in D27 tumours compared with C6 DDAH ± DOX tumours (group A), whereas necrosis was similar in the C6 DDAH tumours (group A) independently of DDAH I expression levels (Figs. 5b, 6c). The endothelial cell content (CD31) of D27 tumours was significantly higher (3.3%) than in C6 DDAH tumours (group A), which had an endothelial cell content of 1.7% in both the absence and presence of DDAH I expression (Figs. 5c, 6d). D27 tumours had a significantly higher perivascular cell content (α-SMA, 3%) compared with C6 DDAH ± DOX tumours (group A) (0.6%) (Figs. 5b, 6e). The fraction of mature vessels, quantified using the ratio of α-SMA stained area versus the CD31 stained area, was also higher in D27 tumours compared with C6 DDAH ± DOX tumours (group A) (Fig. 6f). However, no differences were observed in the C6 DDAH tumours (group A) in the absence or presence of DOX for either CD31 or α-SMA staining.

## Discussion

In this study, a novel tetracycline-inducible DDAH I overexpression system, in which DDAH I was overexpressed in the absence of DOX, was generated from nitric oxide-deficient C6 parental cells, in order to investigate the direct, NO-independent role of DDAH I on tumour growth and angiogenesis. Our key findings indicate that in the absence of NO production, DDAH I overexpression had no effect on tumour growth, tumour vascular characteristics as assessed by in vivo MRI, ex vivo quantification of VEGF, vessel perfusion, hypoxia, endothelial and perivascular cell content, or necrosis.

Previous studies have shown that increased DDAH activity leads to degradation of the NOS inhibitors ADMA and l-NMMA, and therefore increases NO. This has been demonstrated in constitutive DDAH I overexpressing D27 cells and tumours, where increased DDAH I activity reduced ADMA concentration and resulted in a twofold increase in NO synthesis [5]. In our study, the DOX-inducible C6 DDAH cells and tumours (groups A and B) exhibited very limited NO production independently of DOX, and therefore independently of DDAH I expression. D27 tumours produced a significantly higher concentration of NO compared with the C6 DDAH tumours (± DOX). Overexpression of DDAH I in D27 tumours led to faster growth rates compared with wild-type C6 tumours [5]. In our study, the growth rates of the NO-deficient C6 DDAH tumours (groups A and B) were independent of DDAH I overexpression. Hence, in light of our findings, we can deduce that the effect of DDAH on D27 tumour growth reported by Kostourou et al. [5] was NO-mediated. No significant differences were observed between the DOX-treated and untreated C6 DDAH tumours in the MRI measures of fBV and carbogen-induced *R*_2_* changes or in the histologically assessed vessel perfusion, hypoxia, micro-vessel density and vessel maturation. This was probably due to the limited NO production in these tumours. However, in D27 tumours, where production of NO was significantly higher, perivascular cell coverage, and thus vessel maturation, were higher compared with the C6 DDAH tumours (group A). DDAH I protein expression had no effect on the resulting degree of necrosis in C6 DDAH tumours (group A), while D27 tumours, which had high NO production, were more necrotic. Previous studies have shown that high concentrations of NO can directly stimulate apoptosis or necrosis [27, 28]. In C6 DDAH tumours, in the absence of NO, altering DDAH I expression in order to regulate ADMA did not have an effect on the vascular growth of those tumours. We can therefore conclude that DDAH I overexpression alone was not sufficient to overcome the effects of low NO production on tumour growth and vascular development. This is in agreement with a previous study showing that antisense iNOS expressing and DDAH I overexpressing C6 tumours (referred to as ASD10) had similar growth rates and vessel perfusion compared with AS7 tumours (antisense iNOS expressing C6 tumours), and slower growth rates and lower perfusion than D27 (DDAH I overexpressing) and wild-type C6 tumours [29].

The DDAH I/ADMA pathway is known to regulate VEGF-induced angiogenesis in an NO-dependent manner [30, 31]. In the present study, VEGF expression was significantly greater in D27 tumours compared with C6 DDAH tumours (groups A and B) and this correlated with increased NO production in D27 tumours. Increased VEGF expression in D27 tumours (DDAH I overexpressing C6 tumours) has previously been reported in vitro and in vivo [5]. This increased VEGF expression, downstream of an increase in NO production, was diminished in C6 tumours overexpressing a mutant inactive DDAH I enzyme [32]. However, ADMA and DDAH can also regulate VEGF expression via NO-independent mechanisms, such as via DDAH binding to protein kinase A (PKA) and subsequent phosphorylation of the transcription factor Sp1 [10]. In our study, DDAH I overexpression in C6 DDAH cells and tumours and NO accumulation in wild-type C6 cells did not have an effect on VEGF expression, suggesting that in C6 tumours VEGF production is not reliant only on the production of NO. This finding was supported by the fact that in both C6 DDAH and C6 parental cells VEGF was expressed despite the limited NO concentration. Yang et al. [33] suggested that in C6 cells VEGF expression was adjusted and maintained at a certain level according to factors, such as MMPs, HIF-1 and pH. Overall, our study and previous studies suggest that C6 cells can exert both NO-dependent and NO-independent regulation of VEGF.

DOX led to an in vitro increase in VEGF in all cell lines in this study in the absence of cytokine stimulus. However, the effect was diminished in the presence of cytokine and LPS stimulus. Moreover, no similar effect of DOX on VEGF production was observed in the tumour microenvironment in the in vivo model. There are studies that have reported both positive and negative effects of DOX on VEGF and angiogenesis; however, the mechanism of this interaction still remains elusive. A study in murine melanoma tumours has shown that doxycycline treatment increased VEGF expression in vivo, possibly due to aggravation of hypoxia [34]. On the contrary, Su et al. have shown that doxycycline inhibits VEGF-induced angiogenesis in vivo [35]. In our study, the effect of DOX on VEGF does not impinge on our major findings, since this effect was only observed in vitro not in vivo and mainly in the absence of cytokine and LPS stimulus.

We observed that DDAH I activity was significantly lower in D27 tumours compared with C6 DDAH − DOX tumours (groups A and B), despite them having similar levels of DDAH I expression. Leiper et al. [36] have shown that in situations of oxidative or nitrosative stress, NO can inhibit bacterial DDAH enzymatic activity by reversible *S*-nitrosylation of the active-site cysteine (Cys249) of the Cys–His–Glu catalytic triad. This provides a homeostatic mechanism to regulate NO; increased NO production reduces DDAH activity, which in turn leads to ADMA accumulation and inhibition of NOS, thereby restraining further NO production [36]. Therefore, the observations in the present study that D27 tumours showed (a) reduced DDAH activity, and (b) increased NO production could be explained by this negative feedback loop in which NO controls DDAH activity. Kostourou et al. [5] have shown that DDAH activity, assessed by conversion of ^14^C-labelled l-NMMA to ^14^C-labelled citrulline, was high in D27 tumours, suggesting that the time point during tumour growth at which DDAH activity is measured is of great importance. Thus, another explanation for the reduced DDAH activity in the D27 cells observed in the present study could be that at the time of tumour excision (the time point at which DDAH activity was assessed), the amount of DDAH enzyme required to metabolize all the available ADMA was being used, whereas the remainder unused enzyme became inactivated as it was not required.

We have now shown that in C6 gliomas in the absence of NO, endogenous DDAH I overexpression did not alter tumour growth or vascular development. Our data reinforce the hypothesis that regulating DDAH I expression in gliomas by using a DDAH inhibitor could be a useful anti-cancer therapeutic approach, as it could lead to indirect anti-tumour and anti-angiogenic effects by raising endogenous levels of ADMA leading to a subsequent inhibition of NO synthesis. PD 404182, an irreversible inhibitor of human DDAH I, has previously shown therapeutic potential in septic shock-induced vascular collapse. PD 404182 led to ADMA-mediated reduction of NO and abrogated the formation of tube-like structures by endothelial cells in an in vitro angiogenesis assay [37]. Another potent inhibitor of human DDAH I, Cl-NIO, has been shown to decrease NO production in A375 melanoma cells [38]. Such inhibitors appear to be promising tools for the control of methylarginine-mediated NO and could be used as anti-angiogenic therapeutic compounds in diseases where NO production is elevated, such as septic shock, or in diseases characterized by pathologically excessive angiogenesis, such as different types of cancer. Our study provides a rationale for developing novel DDAH inhibitors, such as arginine analogue 10a [39], to control NO biosynthesis and target tumour angiogenesis.

## Electronic supplementary material

Below is the link to the electronic supplementary material.

Supplementary material 1 (PDF 301 KB)
