# Supplementary material for: Assessment of the direct effects of DDAH I on tumour angiogenesis in vivo
Source: Angiogenesis. 2018 May 2;21(4):737–49. doi: 10.1007/s10456-018-9617-6 (PMC6208893; doi:10.1007/s10456-018-9617-6)
Supplement: Supplementary file 1 — Supplementary material 1 (PDF 301 KB) [file 10456_2018_9617_MOESM1_ESM.pdf]

Assessment of DDAH I NO-independent effects on the vascular phenotype of C6 gliomas *in vivo*

Angiogenesis

Efthymia Papaevangelou, Jessica K.R. Boult, Guy S. Whitley, Simon P. Robinson, Franklyn A. Howe

Correspondence to: Efthymia Papaevangelou, School of Immunology and Microbial Sciences, Guy's Hospital, King's College London, London, UK. E-mail: efthymia.papaevangelou@kcl.ac.uk

Electronic Supplementary Material

a

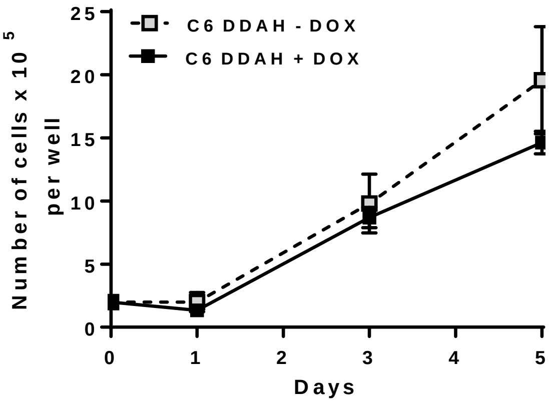

b

Days	Dead cells (%)	
	−DOX	+DOX
1	9.2 ± 1.7	9.0 ± 3.1
3	1.2 ± 0.7	0.6 ± 0.1
5	0.5 ± 0.7	1.2 ± 0.6

c

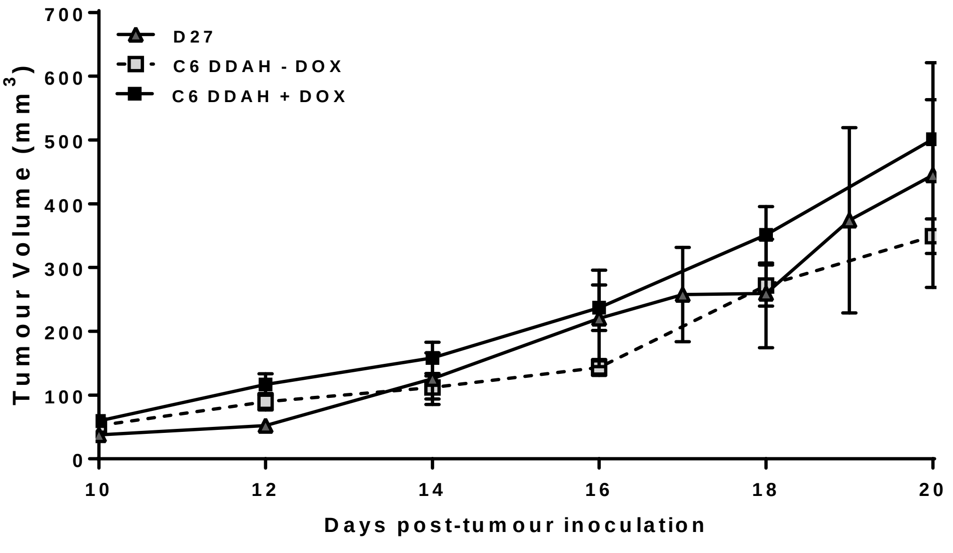

Supplementary Fig. 1 DDAH I effect on cell proliferation and tumour growth in C6 DDAH cells and xenografts (group A). (a,b) Cells seeded at a density of $2x10^5$ cells / well, were treated with 2 µg/ml doxycycline. At days 1, 3 & 5 cells were trypsinised, collected and mixed with eosin, which was taken up by dead cells, and counted. (a) Cell proliferation and (b) percentage of dead cells. Results are the mean ±1 SEM of three separate experiments performed in duplicate. (c) Mean tumour growth curves for C6 DDAH ± DOX (*n* = 6 per group) and D27 (*n* = 4) tumours up to day 20 post-tumour inoculation, when all mice were still alive.

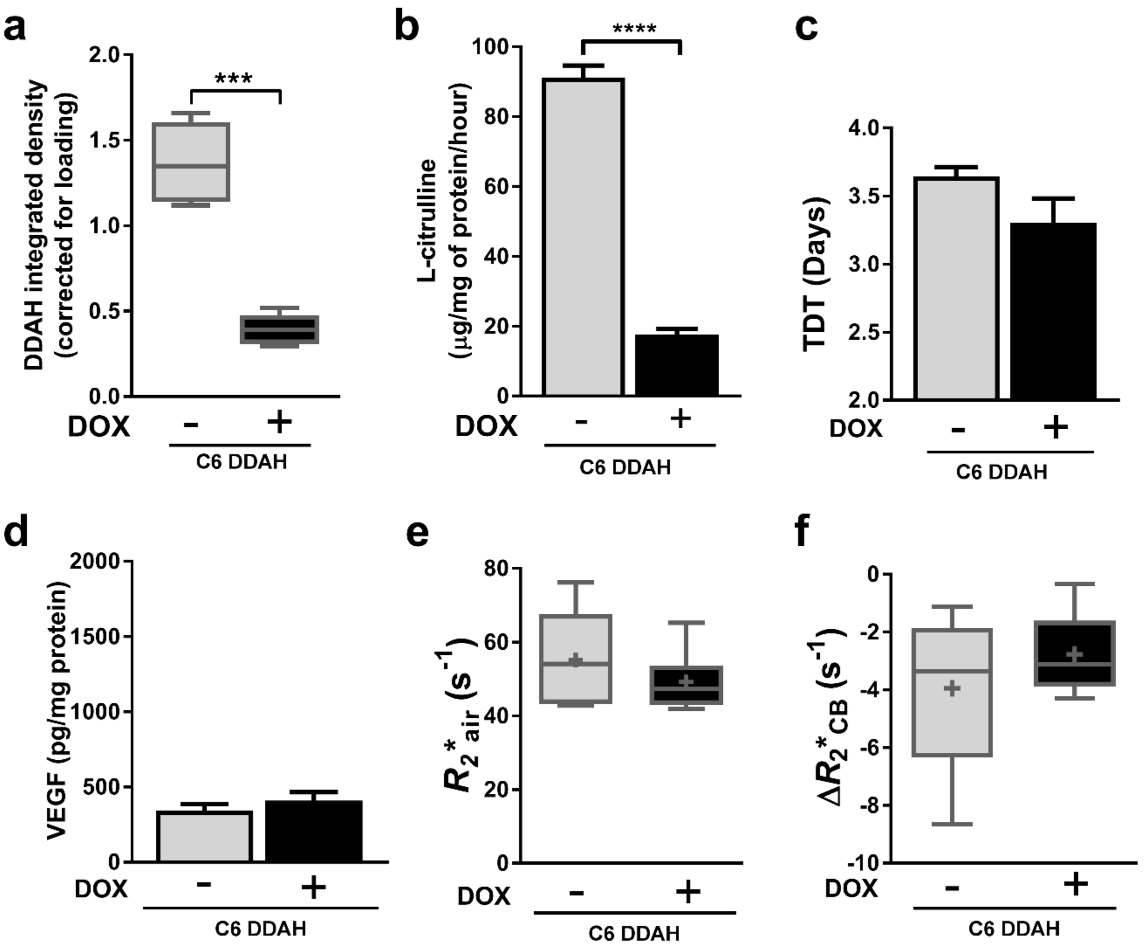

Supplementary Fig. 2 *In vivo* characterisation of C6 DDAH tumour xenografts (group B). C6 DDAH cells were grown in normal medium without doxycycline (group B). Mice with C6 DDAH tumours were given 0.2 mg/ml doxycycline in 5% (w/v) sucrose or 5% (w/v) sucrose alone (n = 6 per group) in the drinking water *ad libitum*. (a) Mean box plot distributions of the integrated densities of the individual protein bands for DDAH I corrected using α-tubulin determined by western blot. (b) L-citrulline production. (c) Tumour doubling times in days. (d) VEGF expression in tumour homogenates. (e,f) Box plot distributions of the median $R_2{*}_{air}$ (e) and $\Delta R_2{*}_{CB}$ (f) values measured by MRI over the whole tumour volume of C6 DDAH ± DOX (group B) (n = 6) xenografts. Results are the mean ± 1 SEM of measurements made from all tumours in each group (***P <0.001, ****P < 0.0001, Unpaired student's t-test).

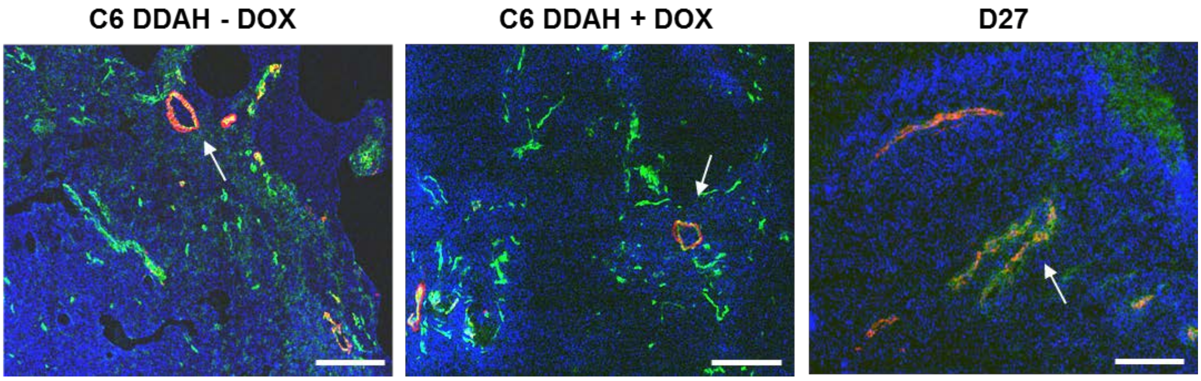

Supplementary Fig. 3 Representative images of areas of tumour sections derived from C6 DDAH ± DOX (group A) and D27 xenografts stained for CD31 and α-SMA. Composite images from C6 DDAH ± DOX and D27 tumour sections stained with the endothelial cell marker CD31 (green fluorescence), the perivascular cell marker α-SMA (red fluorescence) and the nuclear counterstain DAPI (blue fluorescence). White arrows indicate large vessels were the perivascular cells surround the endothelial cell layer. Scale bar is 100 μm.
